# When the Going Gets Tough: A Case Report and Review of Calcinosis Cutis in an Infant with Pseudo-Hypoaldosteronism

**DOI:** 10.7759/cureus.47579

**Published:** 2023-10-24

**Authors:** Nisha Toteja, Daisy Khera, Priya Gupta, Aliza Mittal, Kuldeep Singh

**Affiliations:** 1 Pediatrics, All India Institute of Medical Sciences, Guwahati, IND; 2 Pediatrics, All India Institute of Medical Sciences, Jodhpur, IND; 3 Pediatrics and Public Health, All India Institute of Medical Sciences, Jodhpur, IND

**Keywords:** calcium deposit, iatrogenic calcinosis cutis, intravenous, infusion, pseudo-hypoaldosteronism, calcium gluconate

## Abstract

Calcium gluconate solutions are an essential part of the intensive care medication armamentarium. Calcium-related extravasations are not an infrequent occurrence. However, occult extravasation presenting solely as an isolated mass lesion with no preceding cutaneous manifestation is rare. Calcinosis cutis is an extraosseous collection of calcium deposits in the skin and subcutaneous tissues. Multiple etiopathogenetic factors play a role in its manifestations. We illustrate a case of a seven-week-old infant diagnosed with pseudo-hypoaldosteronism with a mysterious swelling on the left leg during the third week of hospitalization, which was attributed to occult iatrogenic calcinosis cutis.

## Introduction

Extravasation injuries are among the most common iatrogenic morbidities in hospitalized patients [1]. Extravasation is the inadvertent extra venous administration of a medication or solution into the surrounding tissues with the potential for severe tissue or cellular damage [2]. The reported incidence of extravasation injuries with non-vesicant medications is nearly 11% in pediatric patients compared to 0.1-6% in adults [3].

Intravenous calcium infusions have garnered considerable disrepute over the years due to their local complications. A well-recognized complication with its usage is its propensity for extravascular leaks and ensuing tissue damage. The commonly used intravenous calcium-containing solutions, such as calcium gluconate and calcium chloride, possess an osmolality of 669 and 2040 mOsm/L, respectively [4]. The hyperosmolarity combined with the cationic properties of calcium has the potential to cause deep penetrating tissue trauma.

Fortunately, the damage is usually acute, painful, and visible, mandating remedial measures to prevent further injury. Occasionally, minor extravasations may be subtle and go unnoticed. It may rarely transform into a calcified mass or nodular lesion called iatrogenic calcinosis cutis [5]. Pathologically, these extraneous calcium deposits contain hydroxyapatite crystals, which result from a combination of calcium with the exposed collagen in tissues. In vitro, calcium chloride has a more dissociative tendency; however, in practice, calcinosis cutis has been documented with both types of salts.

Clinically, it may mimic conditions such as osteomyelitis, cellulitis, abscess, or thrombophlebitis. In addition, the delayed appearance of this rare lesion makes the temporal correlation between antecedent injury and the ensuing lesion relatively tenuous.

## Case presentation

A term male infant weighing 2810 grams born by normal vaginal delivery was brought to the pediatric department with a history of poor feeding, vomiting, and lethargy for the last two weeks. His admission weight was 2670 grams. On examination, his heart rate was 120/min, respiratory rate was 48/min, oxygen saturation was 99% on room air, blood pressure was 84/50 mm Hg, and random blood sugars were 110 mg/dl. The detailed examination did not reveal any dysmorphism or abnormal genitalia. Serum potassium was 8.21 mmol/L (normal: 3.5-5.1 mmol/L). Serum calcium was 8.5 mg/dl (normal: 8-11 mg/dl). An electrocardiogram was performed, which showed changes in hyperkalemia with tall T waves. His urine test was negative for blood leukocytes and nitrites. He was given supportive therapy for hyperkalemia with calcium gluconate intravenous solution, insulin dextrose solutions, and potassium binding resins. Given persistent hyperkalemia, other treatments for hyperkalemia, such as peritoneal dialysis, were also added later on. A full sepsis workup was performed, which was negative. In addition, 17-OHP (17-hydroxyprogesterone) and serum aldosterone levels were also normal. Thus, the patient was admitted to the pediatric intensive care unit on day two of admission with a presumed diagnosis of pseudo-hypoaldosteronism for correcting life-threatening dyselectrolytemia. High doses of intravenous 10% calcium gluconate (200 mg/kg/day) were given by direct intravenous injection into the peripheral veins during the first two weeks of hospitalization to treat persistent hyperkalemia with ECG changes.

**Figure 1:**
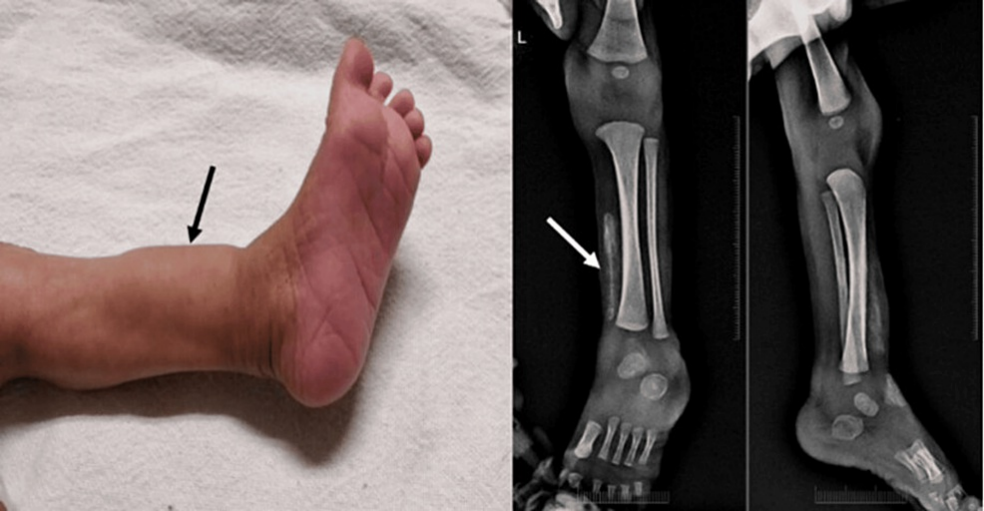
(a) Firm, indurated swelling over the dorsal aspect of the left leg in proximity to the previous cannulation site. (b) Anterior-posterior and lateral view radiographs depicting soft tissue calcifications in the left lower limb

On day 50 of his hospital stay, a swelling was noticed over the left leg. The lesion was hard, non-tender, and gradually progressive (Figure 1a), with no visible signs of inflammation. There was no intravenous catheter in the vicinity of the lesion at this time. Possibilities of cellulitis, osteomyelitis, or abscess were excluded based on appropriate investigations and clinical course. Skeletal radiographs demonstrated radio opacities in the extraosseous space suggestive of calcinosis cutis (Figure 1b). The retrospective review revealed an intravenous catheter in situ in the same limb 10 days back. There was no history of frank extravasation. Investigations revealed normal calcium, phosphorous, and vitamin D levels. His hormonal analysis was within normal limits, including parathormone, cortisol, and aldosterone levels (Table 1). It was, thus, attributed to occult extravasation with delayed presentation as an iatrogenic calcinosis cutis. Spontaneous recovery was documented within four months of its appearance. The genetic basis of pseudo-hypoaldosteronism was later established by confirmatory mutational analysis in the child.

**Table 1:** Hematological and biochemical investigations of the child during the hospital stay

Laboratory parameters	Patient value (normal values)
Haemoglobin	10.7 (11-14 g/dl)
Platelets	243 (150-400x10^9^/L)
Total white cell count	8.8 (4.3-10.8x10^9^/L)
Sodium	114 (136-146 mmol/L)
Potassium	8.21 (3.5-5.1 mmol/L
Chloride	90 (101-109 mmol/L)
Urea	40 (17-43 mg/dl)
Creatinine	0.22 (0.16-0.39 mg/dl)
C-reactive protein	12.26 mg/day (<1)
Calcium, phosphorous, and alkaline phosphatase	9.69 (9.0-11 mg/dL), 4.45 (4-7 mg/dl), and 255 (82-383 U/L)
Intact parathormone	16.7 pg/mL (13.7-52.2 pg/mL)
25-OH vitamin D	64.4 ng/ml (30-100 ng/ml)
Cortisol	15.94 (2-11 µg/dl)
17-OH progesterone	8.13 (3-90 ng/dl)
Serum aldosterone	171.1 (2.52-39.2 ng/dl)
Renin	6.11 (0.15-2.33 ng/ml)

## Discussion

Calcinosis cutis is associated with hypoparathyroidism, hyperphosphatemia, leukemia, connective tissue disorders, trauma, and renal insufficiency [5]. However, iatrogenic calcinosis cutis associated with calcium-containing medications is an unusual occurrence. The first report of soft tissue calcification after an intramuscular calcium injection in an infant was published in 1936 by Tumpeer et al. [5]. Following this, several similar reports emerged, leading to the change in the route of administration from intramuscular to intravenous in the late 1940s. However, the 1970s witnessed reports of complications from intravenous calcium gluconate infusions. Berger et al. published the first series documenting this phenomenon [6]. Goldminz et al. reported a higher incidence of adverse effects related to calcium infusions in premature infants [7].

In a systematic review of calcium infusion-related complications, it was observed that the most common site of lesions was the dorsum of the hand (42%), followed by the upper limb (20%) and the lower limb (18%). The two most frequent symptoms were the appearance of erythema (65%) and swelling/edema (48%), followed by the appearance of skin necrosis (47%), indurated skin (33%), and yellow-white plaques or papules (33%). In nearly two-thirds of reported cases, calcium gluconate was the causative agent. The average volume of calcium infused was 19.2 ml (median 4.9 ml) [2]. A recent report by Soon et al. [8] observed calcinosis cutis in two infants as a complication of parenteral calcium gluconate therapy in the postoperative period. Although not much information is available in published reports regarding cumulative doses of calcium administered and incidence of iatrogenic calcinosis, our index case received three to four doses of intravenous calcium, each of 200 mg/kg, on the first 10 days of admission.

Pathologically, it can be classified into four types: dystrophic, metastatic, idiopathic, and iatrogenic [9]. The most common variety is dystrophic, resulting from systemic pathologies such as infection, inﬂammatory processes, cutaneous neoplasm, or connective tissue diseases. Metastatic calcinosis occurs in the setting of hypercalcemia, causing tissue deposition. The idiopathic term is used when neither local tissue trauma nor systemic metabolic insults can explain these extraneous calcium deposits. The least common entity is iatrogenic calcinosis. Some reports indicate that repetitive heel stick injuries in neonates can give rise to calcified nodules, pathologically pure dystrophic varieties [3].

The pathogenesis of this iatrogenic calcinosis cutis is multifactorial. It is thought to result from a combination of transiently elevated local concentrations of calcium and tissue damage at the extravasation site. However, overt extravasation is not a prerequisite for tissue calcification. Some key factors are the friability of delicate tissues in a small infant, alkaline pH, and an infusion with the propensity for tissue trauma [10]. Concomitant administration of sodium bicarbonate to treat hyperkalemia may have been an aggravating factor in our index case. Other medications exacerbating calcium deposition are prednisolone, sodium phosphate, prochlorperazine maleate, streptomycin sulfate, and amphotericin [7].

Interestingly, calcium infusions are characteristically transparent in vitro and remain inapparent radiologically in the subsequent few days following extravasation. One proposed explanation is that traumatized tissues promote calcium influx and enhance the retention of phosphates bound to the intracellular proteins. This leads to the crystallization of calcium phosphate, which is radiopaque. This process evolves and becomes clinically and radiologically apparent in nearly two weeks. The average time interval documented in the literature is 13 days (two hours - 24 days) [11]. Likewise, this was observed in our case, where the last calcium infusion was administered nearly 10 days before the onset of the calcinotic lesion. A recently published work established a chronogram for extravasation injuries following calcium infusions. They reported that erythema and inflammation appear in the first week, and nodular lesions tend to occur between the first and four weeks. Papules or plaques are reportedly present during the first four weeks, and necrosis occurs within three weeks.

Differential diagnoses include cellulitis, osteomyelitis, arthritis, abscess, periostitis, myositis ossificans, and thrombophlebitis. Simple skeletal X-rays can delineate the characteristic findings of calcinosis cutis and help exclude other competing diagnoses. Lee and Gwinn’s classification describes three types of radiological appearance of calcification following calcium extravasation. The most commonly reported is Type 1, followed by Type 2 and Type 3 [12]. Type 1 pattern is an amorphous mass close to the injection site, resembling myositis ossificans or periostitis. Type 2 is characterized by diffuse calcification in subcutaneous plaques. Lesions resemble calcifications seen in fat necrosis or juvenile dermato-myositis. Type 3 pattern has vascular and perivascular calcification distribution simulating the appearance of arteriosclerosis. Radiographs in our index case suggest subcutaneous plaque-like deposits resembling a Type 2 pattern.

The natural course of iatrogenic calcinosis cutis is usually benign and self-limited. Spontaneous resolution occurs due to trans-epidermal elimination but may take three to six months. Conservative therapies included local cooling and sometimes local heat application. Selective cases with secondary complications may require surgical intervention. The literature reports the use of antidotes such as hyaluronidase in acute calcium extravasations. Hyaluronidase has been successfully used to treat other extravasated substances such as 10-50% dextrose, TPN, calcium, radiographic contrast media, potassium, mannitol, aminophylline, and nafcillin. However, no standardized protocol for the management of calcinosis cutis exists. There are several emerging therapies for calcinosis, depending on the primary cause. Garcia et al. recently reported a case of iatrogenic calcinosis cutis successfully treated with topical sodium thiosulfate [13].

## Conclusions

While meticulous vigilance when using calcium-containing solutions in children remains the best form of prevention, certain safeguards must be exercised. These include switching to oral supplements as early as possible, ensuring flow rates of <2 ml/min, and avoiding co-administration of anions such as bicarbonate, phosphates, and sulfates. In addition, cannulation site checks and regular checks for the patency of intravenous catheters should be emphasized. Timely recognition of these adverse effects can help formulate wise investigative plans and save unnecessary costs and patient suffering.
